# Dental wear patterns reveal dietary ecology and season of death in a historical chimpanzee population

**DOI:** 10.1371/journal.pone.0251309

**Published:** 2021-05-10

**Authors:** Julia Stuhlträger, Ellen Schulz-Kornas, Ottmar Kullmer, Marcel M. Janocha, Roman M. Wittig, Kornelius Kupczik

**Affiliations:** 1 Max Planck Weizmann Center for Integrative Archaeology and Anthropology, Max Planck Institute for Evolutionary Anthropology, Leipzig, Germany; 2 Konrad Lorenz Institute for Evolution and Cognition Research, Klosterneuburg, Austria; 3 Department of Cariology, Endodontics and Periodontology, University of Leipzig, Leipzig, Germany; 4 Department of Palaeoanthropology, Senckenberg Research Institute and Natural History Museum Frankfurt, Frankfurt am Main, Germany; 5 Department of Palaeobiology and Environment, Institute of Ecology, Evolution, and Diversity, Goethe University Frankfurt, Frankfurt am Main, Germany; 6 Max Planck Institute for Evolutionary Anthropology, Leipzig, Germany; 7 Taï Chimpanzee Project, CSRS, Abidjan, Ivory Coast; Ecole Normale Supérieure de Lyon, FRANCE

## Abstract

Dental wear analyses have been widely used to interpret the dietary ecology in primates. However, it remains unclear to what extent a combination of wear analyses acting at distinct temporal scales can be beneficial in interpreting the tooth use of primates with a high variation in their intraspecific dietary ecology. Here, we combine macroscopic tooth wear (occlusal fingerprint analysis, long-term signals) with microscopic 3D surface textures (short-term signals) exploring the tooth use of a historical western chimpanzee population from northeastern Liberia with no detailed dietary records. We compare our results to previously published tooth wear and feeding data of the extant and continually monitored chimpanzees of Taї National Park in Ivory Coast. Macroscopic tooth wear results from molar wear facets of the Liberian population indicate only slightly less wear when compared to the Taї population. This suggests similar long-term feeding behavior between both populations. In contrast, 3D surface texture results show that Liberian chimpanzees have many and small microscopic wear facet features that group them with those Taї chimpanzees that knowingly died during dry periods. This coincides with historical accounts, which indicate that local tribes poached and butchered the Liberian specimens during dust-rich dry periods. In addition, Liberian females and males differ somewhat in their 3D surface textures, with females having more microscopic peaks, smaller hill and dale areas and slightly rougher wear facet surfaces than males. This suggests a higher consumption of insects in Liberian females compared to males, based on similar 3D surface texture patterns previously reported for Taї chimpanzees. Our study opens new options for uncovering details of feeding behaviors of chimpanzees and other living and fossil primates, with macroscopic tooth wear tracing the long-term dietary and environmental history of a single population and microscopic tooth wear addressing short-term changes (e.g. seasonality).

## Introduction

The diet of chimpanzees (*Pan troglodytes*) is highly variable, and includes fruits, leaves and other vegetative plant parts as well as animal resources [e.g. 1–5]. Specific dietary proclivities vary by chimpanzee subspecies, population, and/or social structure [3, 5–8]. For example, *P*. *t*. *troglodytes* of the Lopé Reserve (Gabon) feed more on fruits and less on leaves compared to a population of the same subspecies from the Goualougo Triangle (Democratic Republic of Congo) [2, 9–11]. Moreover, dietary preferences even differ between neighboring communities of the same population, as was recently published for two western chimpanzee (*P*. *t*. *verus*) communities of the population from the Taї National Park (Ivory Coast) [12]. Both communities feed mainly on fruits and other plant parts, but with varying number of plant species. Furthermore, the north community is more engaged in ant feeding, while the south community feeds less on ants but more on honey and bees [12]. Feeding preferences of chimpanzees are also age and sex specific, and depend upon the seasonal availability of food [13, 14]. Generally, female chimpanzees engage more in nut cracking than males [1, 15], and they more often consume insects while males consume more meat from hunted vertebrates [13, 16]. Additionally, environmental factors such as grit and dust have a strong effect on the feeding ecology of chimpanzees. For example, western chimpanzees of the Taї National Park are recurrently exposed to large amounts of wind-borne dust particles during specific dry periods of the year affecting their chewing and digestive efficiency [13].

Both biotic (food) and abiotic (dust) factors lead to dental tissue loss during mastication, which manifests itself macroscopically by generating complementary wear facets on antagonistic occlusal tooth surfaces and microscopically by forming pits/dales and scratches/furrows on these wear facets [e.g. 13, 17–20]. Therefore, tooth wear analysis has been widely used as a tool for reconstructing diets and environmental changes in living and fossil primates and other mammals, often where primary ecological or environmental data were incomplete or lacking [e.g. 21–26].

For example, occlusal fingerprint analysis (OFA) is used to assess the spatial position, size and distribution of wear facets and informs about occlusal 3D jaw movements [23, 27, 28], i.e. the occlusal power stroke that is divided into the incursive shearing phase I and the excursive grinding phase II [29]. It therefore assesses macroscopic wear changes and provides a longer-term signal of wear, accumulated over several months or years [e.g. 30–32]. For example in a human case study, macroscopic wear patterns of a lifetime raw food vegetarian showed inclined wear facets which the authors related to this specific dietary behavior [23]. Moreover, a more recent study on modern and archaeological human teeth showed that hard and tough dietary items produce flat and large wear facets, while a diet consisting mainly of meat produces steeper wear facets [24]. Thus, these studies suggest that distinct dietary differences result in diverging macroscopic tooth wear patterns.

Three-dimensional (3D) surface texture (3DST) analysis, on the other hand, quantifies various micrometer sized geometric aspects on molar wear facet surfaces, such as surface microfeatures height, orientation, density, and complexity [33]. This technique therefore reveals short-term signals on a dental wear facet, i.e. it provides information on the diet consumed up to the last few days prior to an individual’s death [34–36].

Previous studies showed that there is a correlation between macro- and microscopic tooth wear signals. For instance, studies on Neanderthals have demonstrated that both macro- and microscopic wear signals carry an environmentally driven wear signal [24, 37]. Neanderthals from open environments showed enlarged and steep wear facets that formed in a shearing action during mastication [24], while the microscopical surface textures found on these facets were less complex and heterogeneous [37]. Yet, both analyses indicated an increased reliance upon tough, fibrous food such as meat [24, 37]. In recent feeding experiments on rabbits [19], goats [38], and guinea pigs [39] it has been shown that both increased macroscopic tooth wear and microscopic wear signals (high surface roughness and high mean density of pits and furrows) were positively correlated with the amount, shape and size of internal and external dietary abrasives ingested.

**Fig 1:**
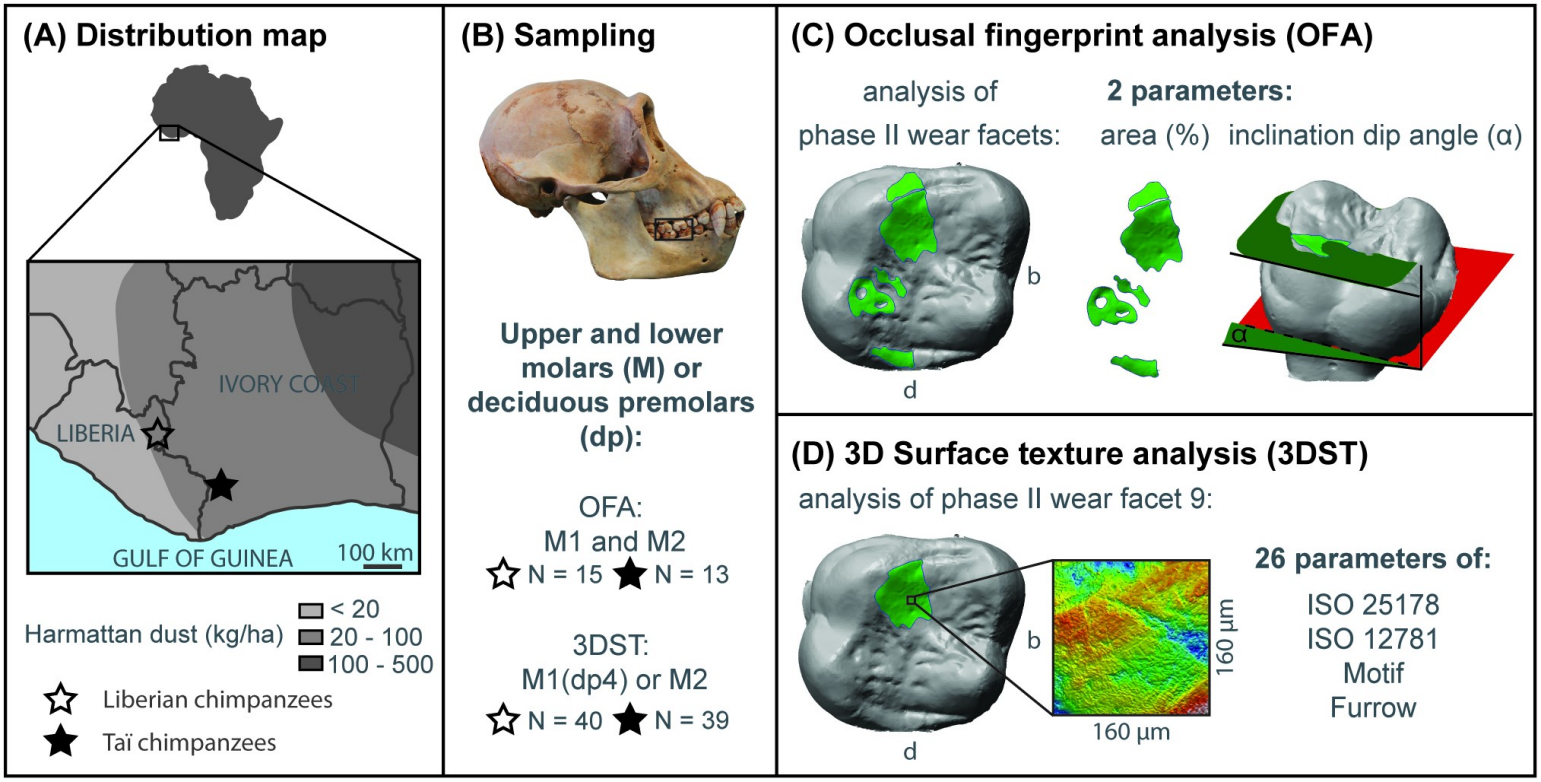
Distribution map and methodological setup. Map with the distribution range of the Liberian chimpanzee population, the locality of the Taї National Park (Ivory Coast) and the distribution of Harmattan dust loads in those areas (A). The map and the Harmattan distribution was redrawn and adapted according to the data presented in Lesschen et al. [45]. Sampling procedure (B) for occlusal fingerprint analysis (macroscopic tooth wear) (C) and 3D surface texture analysis (microscopic tooth wear) (D) shown for the upper first molar (b = buccal, d = distal).

Here we aim to infer the dietary ecology and tooth use of a historical population of western chimpanzees (*P*. *t*. *verus*) from northeastern Liberia for which there are no detailed behavioral and ecological records available. These chimpanzees were poached in the 1950s, and subsequently their skulls were collected and accessioned into the collection of the Senckenberg Research Institute and Natural History Museum Frankfurt. We employ macro- and microscopic wear analyses on both upper and lower first and second molars (M1/M2) as well as deciduous fourth premolars (dp4) and compare the results to those of the extant Taї National Park chimpanzee population whose dietary ecology has been extensively studied since 1979 [1]. Given their geographic proximity in evergreen lowland rain forests [40] (Fig 1A), both populations are assumed to have been exposed to similar climatic and environmental factors such as a bi-annual rainfall pattern, monthly precipitation rate [1, 41] and dust deposition through the Harmattan trade wind during the more pronounced dry periods of a year [42–44]. Using both OFA (long-term macroscopic tooth wear) and 3DST analysis (short-term microscopic tooth wear) we specifically explore (1) whether both populations show similar tooth wear patterns, and (2) whether we can reveal season-specific (dry/rainy) wear patterns within the historical Liberian chimpanzee population, similar to what has previously been found in the Taї chimpanzees [13]. In addition we test for sex-specific differences in tooth wear patterns (both macroscopic and microscopic tooth wear).

## Materials and methods

### Cranial material

The sample of cranial material used in the present study originates from Liberia. It belongs to a large assemblage of chimpanzee skulls housed in the collections of the Department of Paleoanthropology in the Senckenberg Research Institute and Natural History Museum Frankfurt (Frankfurt am Main, Germany, N = 36) and the Phyletic Museum (Jena, Germany, N = 6). In total, we analyzed 100 upper and lower cheek teeth (M1: N = 47, M2: N = 41, dp4: N = 12) of 42 specimens (S1 Table). The crania originate from individuals of a free-ranging western chimpanzee (*P*. *t*. *verus*) population (“Liberian chimpanzees”) in the northeastern part of Nimba County (Liberia). Those individuals were killed, butchered and their skulls usually kept as trophies by locals, called Dan and Kran [46, 47], and later gathered and bought during expeditions led by the German ethnologist and medical doctor Hans Himmelheber in 1952/53 and 1955/56. The individuals originate from a restricted area, which was bounded to the east by the Cess River and to the west by the St. John River. Himmelheber reported the sex of 24 chimpanzees, which was later confirmed by Dierbach [48].

We used a second set of cranial material originating from a free-ranging chimpanzee population of the same subspecies from the Taï National Park (Ivory Coast) to address intraspecific variation in macro- and microscopic tooth wear. This cranial material is part of the osteological collection of the Taï Chimpanzee Project [49] hosted by the Departments of Human Evolution and Primatology of the Max Planck Institute for Evolutionary Anthropology (MPI-EVA Leipzig, Germany). For this population we included 95 upper and lower cheek teeth (M1: N = 49, M2: N = 26, dp4: N = 20) of 41 specimens (S1 Table). Data for the microscopic tooth wear analysis were taken from Schulz-Kornas et al. [13] and Stuhlträger et al. [14].

### Habitat information

The northeastern part of Nimba County (Liberia) and the Taï National Park (Ivory Coast) comprise evergreen lowland rain forest with the latter remaining the largest one in west Africa [1, 40]. Both chimpanzee habitats are in close proximity (approximately 200 km apart, Fig 1A), and underlie similar periodic environmental changes. In the Taï forest the annual rainfall from 1950 to1975 and from 1988 to 1995 was approximately 1800 mm [1], but declined to around 1400 mm annually between 2000 and 2008 (Taï Chimpanzee Project, unpublished data). For Liberia we selected an appropriate location (N6°43’12”, W8°36’36”) and time period (1931–1960) based on historical rainfall data of the Climatic Research Unit of University of East Anglia [41], which documented an average annual rainfall of 1900 mm. In both habitats, the precipitation follows a bi-annual pattern, with two rainy periods (March—June, September—November), separated by months with a decline in rainfall. This decline is strongest from December to February. During this time the rainfall does only vary approximately from 25 mm to 48 mm per month in the Taї forest ([1]; Taï Chimpanzee Project, unpublished data), whereas in northeast Liberia the rainfall does not exceed 42 mm per month (for detailed information on the climate of the Taї forest see [1, 50]). Hence, the period from December to February is considered as a dry period [1]. Furthermore, this particular dry period is accompanied by the Harmattan, a cold desert wind from the Western Sahara, which results in dust deposition in both habitats [42–44].

### Occlusal fingerprint analysis (OFA)

The OFA is a virtual method for the characterization and quantitative comparison of dental macroscopic tooth wear patterns (wear facet pattern) which result from the alteration of the occlusal primary relief in tooth crowns through dental tissue loss during the occlusal power stroke (phase I and phase II) of chewing and other dental activities [23, 27, 51]. A fully developed wear facet pattern in a hominid molar usually consists of a maximum of 13 wear facet positions [52]. Wear facets 1–4 belong to phase I buccal, wear facets 5–8 to phase I lingual, and wear facets 9–13 are attributed to phase II of the occlusal power stroke movements [28]. Here we focused on the upper and lower M1 and M2 complementary wear facets of phase II of the power stroke (Fig 1B and 1C), those wear facets should carry a surface texture induced mainly by food properties, since they are essentially worn through tooth-food-tooth contacts. During chewing, food is trapped, compressed and comminuted between antagonistic phase II facets at the end of phase I and during phase II power stroke movements [53]. To reduce the effect of age and for better comparability we only chose specimens with M1 and M2 in wear stages 3 and 4 according to Smith [54]. Consequently, and compared to the 3DST analysis, we could include only 15 specimens of the Liberian chimpanzees and 13 specimens of the Taї chimpanzees of the OFA study sample.

The OFA uses digital 3D polygonal models of crowns usually generated from high-quality dental casts [55] applying white-light 3D-surface scanning with a spatial minimum point-to-point distance in x-y-z of ~55μm (smartSCAN 3D C-5, Breuckmann GmbH). Polyworks^®^ 2017 (InnovMetric Inc.) 3D metrology software was used for post-processing of the computer models. The Polyworks^®^ Modeler module provides digital tools for a manual segmentation of each wear facets using a close polyline fitted onto the surface of the model while having the original casts under a binocular alongside for a best-practice segmentation [51]. Afterwards the polyline of all wear facets were integrated into the model surface by a re-triangulation of the model surface. Accordingly, wear facet areas were analyzed following the procedures recently described in detail by Kullmer et al. [51]. The OFA method applied here for the wear facet quantification consists of three sequential steps: determination of a reference plane through cervical margin, wear facet segmentation and mapping, and the calculation of wear facet area size and dip angle in respect to the references plane of the molar crown, while fitting a best-fit plane through wear facet surface data points.

The reference plane was calculated from a selection of data points 0.2 mm above and below a manually fitted polyline along the cervical margin (step 1). It was used to orientate the crown model in the Cartesian xy-plane [23, 27, 51, 56, 57]. The closed polyline tool and the grouping of surface data points in Polyworks^®^ 2017 (InnovMetric Inc.) allows the segmentation and labeling of each wear facet (step 2) on the 3D surface model. In the current study the wear facet area size value in mm^2^ and the inclination dip angles of each phase II wear facet are calculated between the reference plane (xy-plane) and the wear facet best-fit plane (step 3). Wear facet areas of phase II facets are calculated as percentage values of the portion of area of wear facets of phase II in respect to the area percentage of all wear facets.

### 3D surface texture (3DST) analysis

The standard phase II wear facet for primate dietary reconstructions [29, 52] is wear facet 9 on both upper and lower molars. In this study, we selected first and second molars on either the right or left side and, where present, of the fourth deciduous premolars. The teeth were cleaned with acetone and molds were taken using a high resolution silicone imprint (Provil^®^ novo Light C.D.2 regular set; Type 3; Heraeus Kulzer, Dormagen, Germany), following the procedure of Schulz et al. [33, 58] and Calandra et al. [25]. We used the high-resolution confocal disc-scanning surface measuring system μsurf mobile (NanoFocus AG, Oberhausen, Germany) with a 100x long distance objective and a numerical aperture of 0.8 to acquire 3DST measurements. Three to four not overlapping scans (each 160 μm x 160 μm) of each facet with a resolution of 0.16 μm in x and y, and a step size of 0.06 μm in z were taken and averaged (mean) for further analyses. Measurements with less than 95% surface points were excluded from further analyses.

The obtained data were analyzed using 26 3DST parameters (Fig 1D, S2 Table) following the 3D areal surface texture standards ISO 25178 [59], ISO 12781 [60] as well as motif (dales and hill recognition using watershed segmentation algorithms) and furrow (surface vectorization) analyses [58] using the μsoft analysis premium software version 7.4.8076 (NanoFocus AG, Oberhausen, Germany; a derivative of Mountains^®^ Analysis software by Digital Surf, Besançon, France). These 3DST parameters relate to criteria such as area (*Sha*, *Sda*, *mea*), density (*Spd*, *Sal*, *medf*), height (*Sq*, *Sp*, *Ssk*, *S5p*, *S5v*, *FLTv*, *FLTp*, *meh*, *madf*, *metf*), volume (*Vv*, *Vvc*, *Vmp*, *mev*), plateau size (*Smr*, *Smc*), anisotropy (*Str*), texture direction (*Std*), complexity (*Sdr*), and slope (*Sdq*).

### Statistics

For all comparisons, we analyzed upper and lower molars separately. For OFA we used both, the first and the second molar, because this method is more affected by the degree of tooth wear. For the 3DST analysis the first permanent molar was the preferred one, i.e. only if this molar was not available, we used the second molar to increase the specimen number. Deciduous fourth premolars were only used for the 3DST comparison of the two populations (dp4_Liberia_ vs. dp4_Taї_) to test whether the possible wear differences already appear in younger individuals. All tests were run between the two populations and between sexes.

We used a circular statistic for OFA to analyze the wear facet dip angles, which we conducted in the software Oriana© (version 3.21, 1994–2010, Kovach-Computing services, Anglesey, Wales, Great Britain) [61–64]. We performed a groupwise Watson-Williams-Test between the mean angles. For the comparison of the phase II wear facet areas, we first checked whether the data are normally distributed. Therefor we used a histogram for visual exploration, as well as the Shapiro-Wilk test that affirmed a normal distribution for our data. Hence, we used the Welch Two Sample t-test in the software R 3.4.3 [65] (using the function “t.test”) to check for statistical significance. The means of wear facet dip angle and area were calculated separately for UM1, LM1, UM2 and LM2.

All the statistical analyses related to the 3DST were performed using the software R 3.4.3 [65] by using the R packages xlsx version 0.4.2 [66], doBy version 4.5.3 [67], and R.utils version 1.12.1 [68]. We used the robust Welch–Yuen heteroscedastic omnibus test (WYT) coupled with a heteroscedastic pairwise comparison test (analog to Dunnett’s T3 test). If both tests detected a significant difference (p = ≤ 0.05) a heteroscedastic rank based test (according to Cliff’s method) was used to detect significant differences between trimmed (15%) means. The trimming method excludes the highest and lowest parts of the data set and is used to compensate for non-normality [25]. For a detailed description of the statistical tests including the R-scripts see Calandra et al. [25, 69].

Additionally, we performed a Factor Analysis (FA) to compare the Liberian chimpanzees with the Taї chimpanzees from dry and rainy periods (for details see S1 Text). We selected 11 of the initial 26 3DST parameters (*Sq*, *Sp*, *Vmp*, *Sdr*, *Sdq*, *Sal*, *Smc*, *meh*, *medf*, *metf*, *mea*) using varimax rotation and the function “factanal”. The rotation “varimax” is recommended for a small sample size and rotates the factors in a way that each variable has a large absolute loading on only one factor, while its loadings on the other factors are close to zero [70]. The 3DST parameters were selected due to approximately normal distribution and no missing values. The Kaiser-Meyer-Olkin measure of sampling adequacy (value > 0.5), using the function “paf” of the R package rela [71], showed that the FA was justified. The FA scores of factors with Eigenvalues ≥1 were extracted to perform a one-way ANOVA with a post-hoc Tukey’s Honestly Significant Difference (HSD) test in order to explore significant variations between groups.

## Results

**Fig 2:**
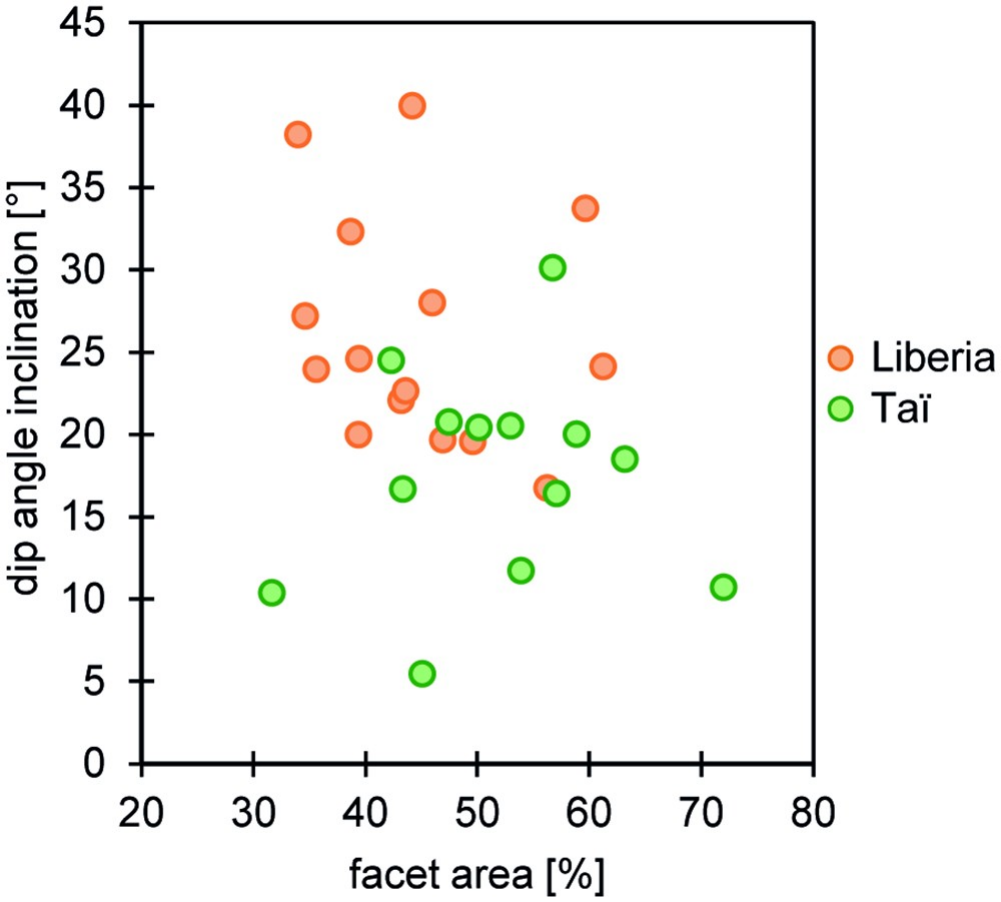
Facet area and dip angles of phase II wear facets of the upper second molars compared between both chimpanzee populations.

The OFA results reveal that both populations differ to some extent in their macroscopic wear pattern; the angle of facet inclination shows a trend towards steeper phase II wear facets (larger angle of inclination) and smaller phase II wear facet areas in the Liberian population of chimpanzees compared to Taï chimpanzees (Fig 2, Table 1). This difference is significant only on the upper second molars (facet inclination: p_Watson-Williams_ = 0.004, facet area: p_Welch Two Sample t-Test_ = 0.047) (Table 1), reflecting differences in the occlusal relief of the molars. Steeper and smaller wear facets point to lower rates of tissue loss in the Liberian chimpanzees indicating differences in chewing activity.

**Table 1:** Descriptive statistics for dip angles and areas for phase II wear facets.

Test variable 1 vs. 2	Teeth		phase II dip angle [°]						phase II area [%]						
		Test variable 1			Test variable 2			p-value^a^	Test variable 1			Test variable 2			p-value^b^
		N	Mean	SD	N	Mean	SD		N	Mean	SD	N	Mean	SD	
*Liberia vs*. *Taї*	UM1	15	21.45	7.4	15	20.77	7.39	0.689	15	39.85	7.72	12	44.71	6.44	0.087
	LM1	14	27.23	5.53	7	25.16	7.27	0.476	14	43.88	8.59	7	49.98	9.82	0.191
	UM2	15	25.57	7.01	13	17.51	6.59	**0.004**	15	44.81	8.06	13	51.89	10.32	**0.047**
	LM2	14	27.62	7.07	9	25.23	4.14	0.368	14	44.71	10.96	9	47.96	7.92	0.419
*Liberian females vs*. *Taї females*	UM1	4	18.85	9.05	7	15.37	4.41	0.406	4	38.9	5.77	7	43.81	3.88	0.194
	LM1	4	26.61	6.75	4	23.73	7.09	0.577	3	41.5	8.98	4	48.03	9.9	0.406
	UM2	4	26.34	9.33	8	14.66	5.82	**0.022**	4	41.05	10.4	8	52.01	11.72	0.144
	LM2	4	27.65	11.81	5	26.83	4.47	0.886	5	51.81	12.15	5	47.06	7.02	0.476
*Liberian males vs*. *Taї males*	UM1	8	21.03	5.38	5	21.15	5.99	0.971	7	38.12	6.42	5	45.97	9.39	0.152
	LM1	7	27.85	4.11	3	27.06	8.59	0.844	6	46.26	5	3	52.58	11.17	0.434
	UM2	8	23.76	4.71	5	22.07	5.37	0.563	7	42.41	4.89	5	51.69	8.89	0.081
	LM2	7	27.53	3.57	4	23.23	3.08	0.076	6	39.73	5.17	4	49.09	9.93	0.156
*Liberian females vs*. *Liberian males*	UM1	4	18.85	9.05	8	21.03	5.38	0.604	4	38.90	5.77	7	38.12	6.42	0.842
	LM1	4	26.61	6.75	7	27.85	4.11	0.711	3	41.50	8.98	6	46.26	5	0.462
	UM2	4	26.34	9.33	8	23.76	4.71	0.528	4	41.05	10.40	7	42.41	4.89	0.819
	LM2	4	27.65	11.81	7	27.53	3.57	0.975	5	51.81	12.15	6	39.73	5.17	0.091
*Taї females vs*. *Taї males*	UM1	7	15.37	4.41	5	21.15	5.99	0.082	7	43.81	3.88	5	45.97	9.39	0.648
	LM1	4	23.73	7.09	3	27.06	8.59	0.596	4	48.03	9.9	3	52.58	11.17	0.604
	UM2	8	14.66	5.82	5	22.07	5.37	**0.042**	8	52.01	11.72	5	51.69	8.89	0.957
	LM2	5	26.83	4.47	4	23.23	3.08	0.214	5	47.06	7.02	4	49.09	9.93	0.744

UM = upper molars, LM = lower molars, N = number of individuals, SD = standard deviation, p = level of significance,

^a^ p-values from Watson-Williams-Test,

^b^ p-values from Welch Two Sample t-Test.

The combination of the Welch–Yuen heteroscedastic omnibus test, a heteroscedastic pairwise comparison test and a heteroscedastic rank based test using a total of 26 3DST variables reveal that both populations (mixed sex) distinctly differ in their microscopic wear pattern (S3 Table) on both upper and lower M1/M2 and dp4. The surface textures of the Liberian chimpanzees M1/M2 and dp4 are significantly flatter (*Sq*, *S5v*, *FLTv*, *FLTp*), less voluminous (*Vv*, *Vvc*), and show a higher density of peaks (*Spd*) and furrows (*medf*) with small hill areas (*Sha*, *mea)* and dale areas *(Sda*) than those of the Taï chimpanzees (S3 Table).

**Fig 3:**
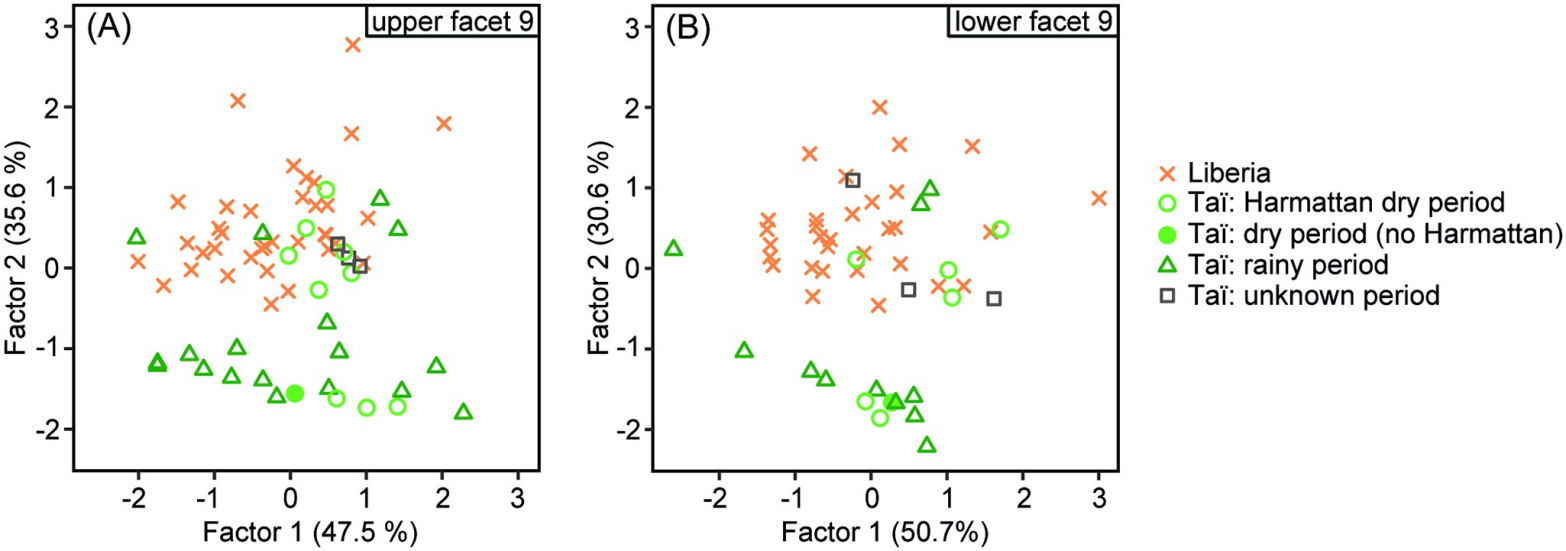
Factor analysis of wear facet 9 comparing the Liberian chimpanzee population to Taї chimpanzees from dry and rainy periods. (A) upper molars; (B) lower molars; 3DST data for Taї chimpanzees taken from Schulz-Kornas et al. [13].

When testing for presence of a seasonality signal, using FA with two factors we found that the 3DSTs of the Liberian upper and lower M1s and M2s overlap with those individuals of the Taï chimpanzees that died during the dry period (upper facet 9: p_Tukey_ = 1, lower facet 9: p_Tukey_ = 0.865) but are significantly different from those that died during the rainy period (upper facet 9: p_Tukey_ = 0.005, lower facet 9: p_Tukey_ = 0.007) (Fig 3, Table 2). For both, upper and lower facet 9, the factor loadings suggest that the height parameters (*Sq*, *Sp*, *meh*, *metf*) as well as the parameters *Vmp* (volume) and *Smc* (plateau size) contribute most to factor 1, whereas *Sdr* (complexity), *Sdq* (slope), *mea* (area) and *medf* (density) contribute most to factor 2 (S4 Table). The separation of Liberian chimpanzees from Taї chimpanzees that died during a rainy period is mainly driven by factor 2 (Fig 3), indicating more complex 3DSTs with a higher density of furrows in the Liberian chimpanzees.

**Table 2:** Post-hoc Tukey’s Test for the scores of factor 1 and 2 of the factor analysis comparing Liberian chimpanzees to Taï chimpanzees from dry and rainy period.

Data set	Test	Mean Difference	Lower CL*	Upper CL*	p-value
upper facet 9	Taï dry (8) vs. Liberia (36)	0.01	-1.24	1.27	1.000
	Taï wet (18) vs. Liberia (36)	-1.26	-2.19	-0.34	**0.005**
	Taï wet (18) vs. Taï dry (8)	-1.28	-2.64	0.09	0.071
lower facet 9	Taï dry (6) vs. Liberia (31)	-0.30	-1.74	1.13	0.865
	Taï wet (11) vs. Liberia (31)	-1.50	-2.62	-0.37	**0.007**
	Taï wet (11) vs. Taï dry (6)	-1.19	-2.82	0.44	0.190

*95% family-wise confidence level (CL), values in brackets indicate the sample size.

**Fig 4:**
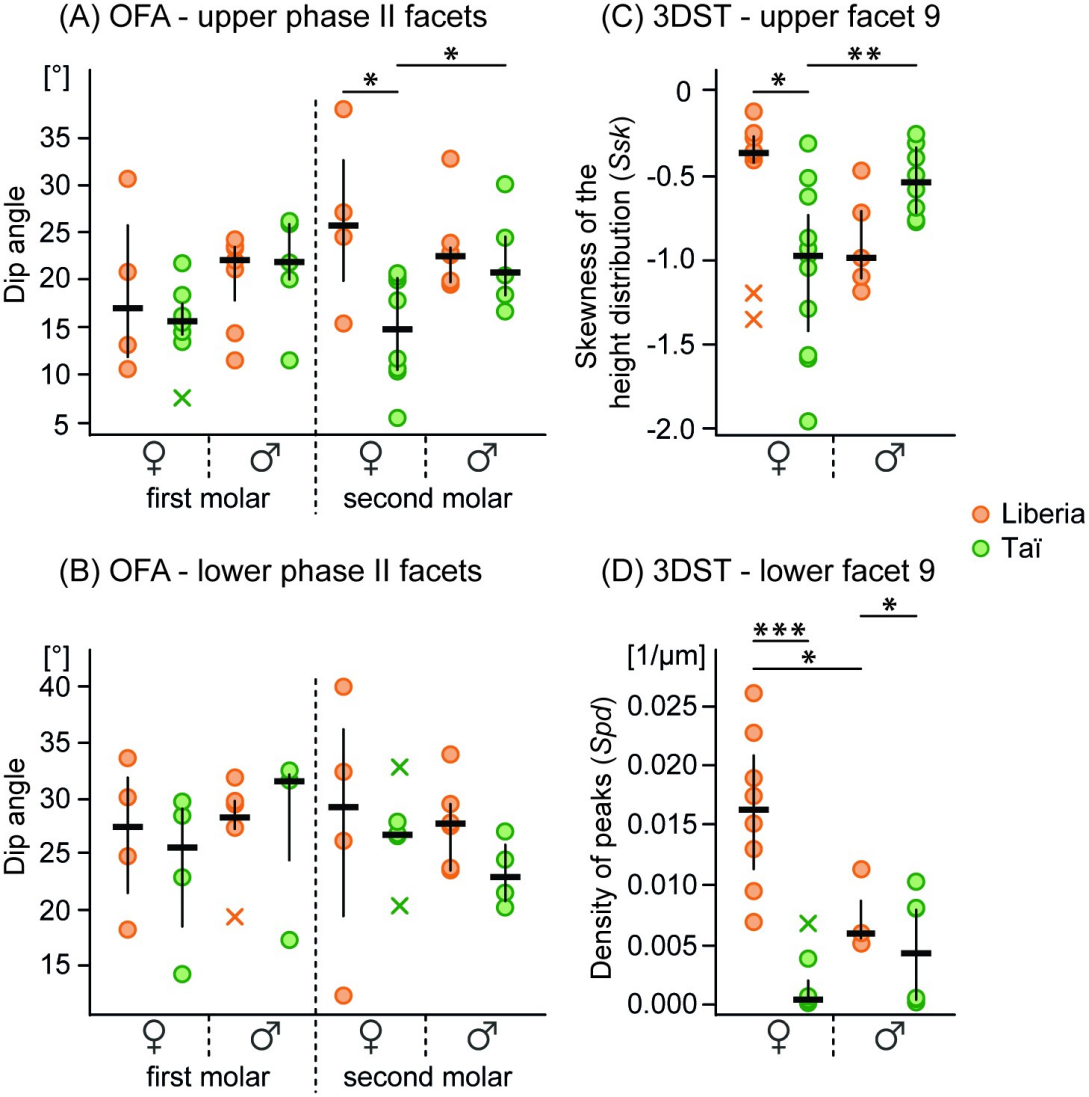
Variation between sexes and populations shown for phase II dip angles and selected 3DST parameters. Dip angle calculation on (A) upper and (B) lower phase II wear facets; (C) skewness of the height distribution (*Ssk*) on the upper facet 9; (D) density of peaks (*Spd*) on the lower facet 9. Strip charts indicate data median = horizontal line, interquartile range (IQR) = vertical line, and outlier (> 1.5*IQR) = x, significance levels are indicated by asterisks (* ≤ 0.05, ** < 0.01, *** < 0.001).

The analysis of a sex-specific wear pattern reveals that female chimpanzees from Liberia have significantly steeper phase II wear facets on their upper second molars than females from Taï (p_Watson-Williams_ = 0.022), while males of both populations exhibit a similar macroscopic tooth wear pattern (Fig 4A and 4B). Liberian females also have slightly smaller phase II wear facet areas compared to Taï females, but none of these differences are significant (Table 1). Furthermore, females and males within both populations do not differ in their phase II wear facet areas (Table 1). Additionally, Liberian and Taï females differ in their 3DSTs to a greater degree than Liberian and Taï males (S5 Table). Specifically, Liberian females have more complex (*Sdr*) surface textures with significantly more peaks and less plateaus than Taï females as indicated on the upper molars by *Ssk* (Fig 4C) and on the lower molars by *Spd* (Fig 4D), *medf* and *Sda* (S6 Table). Males of both chimpanzee populations show greater similarity in their 3DSTs, differing exclusively in the characteristics of peaks (Fig 4D, S5 Table).

## Discussion

Tooth wear analysis is a valuable tool to reconstruct the dietary ecology of fossil and extant primates at the interspecific and intraspecific level. For example, interspecific and intraspecific studies on early *Homo sapiens* and different groups of Neanderthals showed that both macroscopic (long-term) and microscopic (short-term) tooth wear analyses can be informative about dietary (biotic) and environmental (abiotic) factors [e.g. 24, 37]. Therefore, the aim of the present study was to infer the feeding ecology and environmental settings of a historical western chimpanzee population from northeastern Liberia by comparing its macroscopic and microscopic tooth wear patterns to those of a reference sample from the Taï chimpanzee population whose dietary ecology is well established. We specifically asked (1) whether both populations show similar tooth wear signals and thus share a similar feeding ecology given their geographic proximity, and (2) whether any seasonal fluctuations (dry and rainy periods) in the environment and sex-based feeding habits could be detected in the Liberian chimpanzee population. We found that both populations have distinct tooth wear patterns with Liberian chimpanzees having macroscopically slightly steeper dip angles and smaller phase II wear facet areas and microscopically flatter and less rough wear facet surface textures with a higher density of small peaks and furrows than Taї chimpanzees (see Table 1, Figs 2 and 3). This suggests a higher concentration of small internal (e.g. phytoliths) or external (e.g. dust) abrasives in the diet of the Liberian chimpanzees, at least shortly before their death [e.g. 13, 25]. Moreover, and similar to the observed sex-specific differences among Taї chimpanzees, female and male Liberian chimpanzees differ in their microscopic tooth wear patterns. However, at this point these differences need to be considered cautiously due to the limited sample size for male Liberian chimpanzees (5 males for upper facet 9, 3 males for lower facet 9). Most interestingly, however, is the finding that the 3DST analysis groups the Liberian chimpanzees with those chimpanzees from the Taї population that knowingly died during dry periods, while they significantly differ from those Taї chimpanzees that died during rainy periods (see Table 2).

### Inferring environmental abrasives and seasonality from tooth wear

The macroscopic tooth wear patterns of both populations are similar. However, there seems to be a trend towards steeper and smaller wear facets in Liberian chimpanzees compared to Taї chimpanzees, with an exception of the upper M2s where Liberian chimpanzee wear facets are significantly steeper and smaller. Since we exclusively used teeth with similar tooth wear stages (for details see Occlusal fingerprint analysis (OFA) in Materials and Methods) for the macroscopic tooth wear analyses, we do not think that the steeper and smaller phase II wear facets of Liberian chimpanzees can be explained by age differences among individuals of both populations. We rather suggest that steeper and smaller wear facets point to a lower rate of tissue loss in the Liberian chimpanzees, probably as a cumulative result of varying dietary properties dominated by low abrasive (a)biotic particles in a long-term perspective [19]. In contrast, more planar and larger wear facets, which are related to a higher rate of tissue loss, point toward a high abrasive diet. Previous studies on the feeding ecology of chimpanzees from regions around the Nimba Mountains, which are close to the reported distribution area of the Liberian chimpanzees studied here, reported intense consumption of different species of ants [72–74], which are considered as large abrasive dietary particles causing tooth wear [e.g. 25, 75]. In contrast, the Taї chimpanzees are well-known for their use of a hammer (e.g. branches or stones) and an anvil (e.g. root, stone) to access the soft endosperm (low abrasive) of nuts without chewing on the harder nut shells (for details see Supplement 5 in [13]). These particular and detailed dietary behaviors are not reflected in our macroscopic tooth wear results. However, it needs to be considered that first our sample size is quite small for the OFA data, and second the steeper and smaller wear facets in Liberian chimpanzees compared to Taї chimpanzees are statistically non-significant (with an exception of the upper M2) and therefore can only be treated as a trend. Hence, at this stage, it is too early to draw definite conclusions from the OFA, and it needs to be investigated further.

In contrast, the microscopic tooth wear results support the findings of previous studies that the Liberian chimpanzees, as a whole population (both sexes), presumably have fed more on insects (high density of microscopically small hill- and dale-like features) when compared to the Taї population (both sexes), who focused more on the consumption of nut endosperm. The Liberian chimpanzees in our sample show microscopically smoother wear facet surfaces with a higher density of narrow features than the Taї chimpanzees (pooled data set including dry and rainy period). A high density of microscopically small hill- and dale-like features (high values for *Spd*, small values for *Sha*, *Sda*) is indicative for chewing on large and hard dietary items, such as sclerotized insect exoskeletons or seeds [25]. Large hard food items fracture tooth enamel more heavily, and therefore microscopic hill- and dale-like features tend to be smaller since it is unlikely that large ones persist [25].

In addition to the inferences about the feeding preferences of Liberian chimpanzees, we found that the Liberian chimpanzees, with their many small and narrow microscopic wear features, cluster in the Factor Analysis (FA) with the Taї chimpanzees that died during dry periods (see Fig 3) (for details on the 3DST results of the Taї chimpanzees see [13]). Some of the former even have higher values on factor 2 than any of the Taї chimpanzees, implying that these Liberian individuals have the smallest and narrowest microscopic wear features of the whole data set. The habitats of both chimpanzee populations are characterized by a yearly occurring major dry period, approximately lasting from December until February [1, 41]. This dry period is accompanied by a dust-laden trade wind, the Harmattan, blowing from the Sahara Desert over West Africa into the Gulf of Guinea [43]. In fact, we know that the Harmattan reaches the Taї National Park for up to two weeks in this particular dry period, and that the forest is covered with dust particles until they are washed down by the rain at the beginning of the rainy period [1]. Despite the fact that the Liberian chimpanzees cluster with the majority of the Taї chimpanzees from Harmattan dry periods while differing from most of the Taї chimpanzees from rainy periods, both Taї chimpanzees from dry and rainy periods show some overlap in the FA, which may be accounted for as follows: Firstly, some of the Taї chimpanzee specimens died at the very beginning or end of a dry and/or rainy period. Even though the 3DST parameters reveal short-term tooth wear signals, the accumulation of new wear signals may take several days or a few weeks depending on the dietary particles [34–36]. For example, an individual that died at the beginning of the rainy period may still show microscopic wear features related to the prior period, the Harmattan dry period. Secondly, the overlap of Taї chimpanzees from dry and rainy periods might be explained by the fact that the exact arrival of the Harmattan wind in the Taї forest, which we assume as one of the major causes of the microscopic wear signals, might have varied between years (e.g. few days to one week earlier or later). Due to the lack of information of the exact arrival and duration of the Harmattan wind in each year, we used all months of the dry period (December to February) [1] and assigned them to the Harmattan dry period in our analyses.

Despite the overlap of the Taї chimpanzees from the Harmattan dry period with those from the rainy period, the former show more complex 3DSTs with a higher density of furrows compared to those Taï chimpanzees that died during the rainy period. The same applies for the Liberian chimpanzees. This is in accordance with previous studies that showed that the ingestion of abrasive components (e.g. dust) creates complex microscopic tooth wear signals with many and fine furrows and dales [13, 76]. Additionally, tooth wear and fecal particle sizes in Taї chimpanzees showed that during the dry period dust particles are consumed with the food and that their presence is reflected by a specific 3DST pattern on their molars [13]. Based on the similarities in the microscopic tooth wear of the Liberian chimpanzees and Taї chimpanzees that died during Harmattan dry periods as well as the fact that the Harmattan wind has also been present in Liberia at least since the early 20th century [77] it is highly conceivable that the Liberian chimpanzees died during such a Harmattan dry period. The chimpanzees in northeastern Liberia were reported to be under intense human predation [78]. Historical accounts from the 1950s indicate that the Liberian specimens used in this study were poached and butchered by local tribes of farmers, called Dan and Kran, and the skulls were kept as trophies [46, 47]. Even nowadays, hunting for bush meat in Nimba County (Liberia) is still a common activity by local groups; 91% of the people hunt year-round, in dry and rainy periods, but 80% of those people reported to increase their hunting activity intensively during dry periods [79]. This, in turn, is in line with our interpretation that the chimpanzees studied here most likely perished during an annual dry period, which might have accompanied by the dust-laden Harmattan trade wind. However, additional historical or cultural evidence from that time period is needed to reinforce our suggestion.

### Sex-specific feeding ecology in Liberian chimpanzees

It has been widely known that chimpanzees show sex-specific feeding preferences [e.g. 1, 16, 80–82]. The macroscopic tooth wear results suggest that female chimpanzees tend to engage in a different chewing behavior than males. This is more obvious in female Taї chimpanzees where more planar and less inclined phase II wear facet surfaces are indicative of vertical chewing movements. Such chewing kinematics are thought to be required when dietary items need to be crushed (e.g. hard exoskeletons of insects, seeds) [83]. This tooth wear pattern is more pronounced on the upper second molars of Taї chimpanzees where females have significantly smaller phase II dip angles than Taї males. However, the trend for less inclined phase II wear facets in Taї females compared to males is also seen on the other molars (see Table 1). Large and less inclined phase II wear facets of Taї females indicate a high degree of repetitive vertical crushing combined with horizontal grinding activity. This could be related to an intense comminution of items like insects with hard exoskeletons and seeds embedded in fruit pulp (< 1.2 cm), on a regular basis [13].

Recently, Schulz-Kornas et al. [13] showed for the Taї chimpanzee population that female/male-specific dietary differences are reflected in the 3DST pattern. It was found that both sexes within the Taї population consume mainly fruit pulp without any hard seeds, but they differ in their feeding time on other dietary items. Females who consume more fruit pulp including large amounts of small seeds, leaves and insects have microscopically flatter and less voluminous wear facet surfaces with many micrometer-sized plateau-like features [13]. In contrast, males who feed more extensively on large seeds, vertebrate meat, and in contrast to other observations [1, 15], on a larger amount on nut endosperms, have wear facet surfaces dominated by larger dales and less plateaus [13]. However, for the comparison with our data it needs to be considered that the dietary observations of Taї male chimpanzees arise mainly from the Harmattan dry period, which may mean that the dietary and dust signal in their 3DST is mixed [13].

Our results show that Liberian females and males differ to some extent in their 3DSTs, but in a different way, than has been described for Taї females and males. Liberian females have significantly more microscopic peaks and smaller hill and dale areas as well as slightly rougher wear facet surfaces than the Liberian males, although both Liberian females and males have more peaks than Taї chimpanzees of both sexes (see Fig 4D, S6 Table). Since chewing on large and hard dietary items such as sclerotized insect exoskeletons can produce rougher wear facet surfaces and many of the microscopically small hill-like features [25], we conclude that Liberian females consumed more insects than Liberian males. This is in agreement with Smith et al. [80] who analyzed carbon signatures of the Ganta chimpanzees from northern Nimba County (Liberia). This analysis suggested a higher insect consumption in female chimpanzees and a higher meat consumption in male chimpanzees. Although the Taї females are also known to consume more insects than males [13]; the former may have consumed less insects and more softer dietary items (e.g. nut endosperm, fruit pulp) when compared to the Liberian females. Therefore, the 3DST results suggest that both populations have different feeding ecologies, at least on a short-term basis, and that this is mainly driven by the female chimpanzees of each group.

## Conclusions

In our chimpanzee example, the macroscopic tooth wear data (OFA) suggest a similar dietary ecology between both populations on a long-term basis. In contrast, microscopic tooth wear signals (3DST) were able to detect inferences about the season of death of the Liberian chimpanzees by using the Taї chimpanzee population as a reference data set. Furthermore, the 3DST results are robust in pointing out differences between the feeding habits of females and males and between populations, since it represents a direct signal of what the individuals consumed shortly before their death.

Our results therefore highlight that a combination of long-term and short-term dental wear analyses opens new options for uncovering details of ecological and dietary behaviors of chimpanzees, but also of other primates and other animal groups. Working on both scales should therefore be considered in future studies since it allows tracing changes in the long- and short-term dietary and environmental history of a single population.

## Supporting information

S1 Text Additional information about the conducted factor analysis and the R script. (PDF)

S1 Table Specimens sampled for occlusal fingerprint analysis (OFA) and 3D surface texture (3DST) analysis. Wear facet 9 (for 3DST analysis) and phase II wear facets (for OFA) on deciduous fourth premolars (dp4), permanent first (M1) and second (M2) molars of the upper (U) and lower (L) jaw were sampled. (n.a.) no information is available. Taï chimpanzee data including their 3DST data are taken from Schulz-Kornas at el. [13] and Stuhlträger et al. [14]. ^a^ Taï chimpanzee project unpublished data.
(XLSX)

S2 Table Description of the 3D surface texture parameters (3DST) that were selected for this study. (XLSX)

S3 Table Descriptive and test statistics of significant 3D surface texture (3DST) parameters on facet 9 between Liberian and Taï chimpanzees. To test the level of significance a combination of the robust Welch–Yuen heteroscedastic omnibus test (WYT), heteroscedastic pairwise comparison test (Dunnett), heteroscedastic rank based test (Cliff) were used. Following Calandra et al. [25] values in bold indicate a significant differences (p ≤ pc ≤ 0.05), no significant difference was detected in case of p > 0.05 and p ≥ pc). U = upper teeth, L = lower teeth, M1 = first molar, M2 = second molar, dp4 = fourth deciduous premolar, N = number of individuals, SD = standard deviation, Ft / ph = test statistics, p = level of significance, nu1 / nu2 / Df = degree of freedom, pl = lower 95% confidence interval, pu = upper 95% confidence interval, pc = critical significance level adjusted for family-wise error.
(XLSX)

S4 Table Loadings of the factor analysis for the comparison of the Liberian and Taї chimpanzees from dry and rainy periods given for each 3DST parameter and factors with eigenvalues >1. First or second molars of 67 (upper facet 9) and 52 (lower facet 9) individuals were used for the factor analysis, respectively. Values in bold indicate interpretable loadings (≥ 0.7) [70].
(XLSX)

S5 Table Test statistics for 3D surface texture (3DST) comparisons given for significant parameters and sorted according to data set. Following Calandra et al. [25] values in bold indicate a significant differences (p ≤ pcrit ≤ 0.05), no significant difference was detected in case of p ≥ pc. N = number of individuals, UM = upper molars, LM = lower molars, f = facet, Ft / ph = test statistics, p = level of significance, nu1 / nu2 / Df = degree of freedom, pl = lower 95% confidence interval, pu = upper 95% confidence interval, pc = critical significance level adjusted for family-wise error.
(XLSX)

S6 Table Descriptive statistics for 3D surface texture parameters (3DST) of female and male Liberian chimpanzees. N = number of individuals, SD = standard deviation.
(XLSX)
