# Supplementary material for: Dental wear patterns reveal dietary ecology and season of death in a historical chimpanzee population
Source: PLoS One. 2021 May 10;16(5):e0251309. doi: 10.1371/journal.pone.0251309 (PMC8109778; doi:10.1371/journal.pone.0251309)
Supplement: S1 Text — (PDF) [file pone.0251309.s001.pdf]

S1 Text: Additional information about the conducted Factor Analysis and the R script

We decided to run a Factor Analysis (FA) instead of a Principal Component Analysis (PCA) or Cluster Analysis (CA), because it is normally used to primary determine and asses behavioral constructs (in our case the comparison of the seasonality and the populations) [1]. Furthermore, it is unlikely in a FA that low and non-significant correlations produce high FA loadings [1]. In contrast, in a PCA the main objective is to reduce the number of dimensions [1], while a CA presumes that there are no defined groups a priori [2].

The following R script was adapted from Stuhlträger et al. [3].

R script:

```
# Load libraries

library(xlsx)
library(doBy)
library(R.utils)
library(car)
library(devtools)
library(rela)


# Define the modes of the columns in "vect_mode" and import data into "pan"
vect_mode <- c(rep("character",10), rep("numeric",36))
pan <- read.xlsx2(file=file.choose(), sheetIndex=1, header=TRUE, colClasses=vect_mode)

# Define the variables (parameters) according to their column names
vars <- colnames(pan)

# Create a vector with names of the parameters which should be removed from the analysis
entf <- c("Sha", "Sda", "S5p", "S5v", "Str", "Std", "Vv", "madf", "Spd", "FLTv", "mev", "Smr","Vvc","FLTp")

# Remove the parameters and save it in a new data frame
pan_sel <- pan[, !(vars %in% entf)]

# Create a vector with parameters that should be included in the FA
parameters <- c("Sq", "Sp", "Vmp", "Sdr", "Sdq", "Sal","Smc", "meh", "medf", "metf", "mea")

# Create a subset for upper (tx) and lower (tm) teeth[1] as well as facet 9 (f9)
pan.tx <- subset(pan_sel, BONE == "tx" & FACET == "f9")
pan.tx[] <- lapply(pan.tx, function(x) if(is.factor(x)) factor(x) else x)

# Check if the parameters are approximately normally distributed
shap_test <- c()            # Shapiro-Wilk Test: parameters are normally distributed if p ≥ 0.05
shap_vec <- c()
for(i in 1:length(parameters)){
shap_test <- shapiro.test(pan.tx[,parameters[i]])
shap_vec <- rbind(shap_vec, shap_test)}
row.names(shap_vec) <- parameters

# Sq, Smc, metf are normally distributed
```

[1] in the following all descriptions are made for upper (tx) teeth, but the analysis were also applied on lower (tm) teeth

```
par(mfrow=c(4, 4))                                # inspect the distribution of the parameters virtually
histo <- c()
histo_7 <- c()
for(i in 1:length(parameters)){
a=length(histo)
histo <- hist(pan.tx[,parameters[i]], main=parameters[i])
histo_7 = rbind(histo, histo_7)
i+1}
```

Transform parameters that are not normal distributed to reach an appropriate distribution for further analyses

```
pan.txf9$Sp=(log(pan.txf9$Sp))
pan.txf9$Vmp=(log(pan.txf9$Vmp))
pan.txf9$Sdr=(sqrt(pan.txf9$Sdr))
pan.txf9$Sdq=(log(pan.txf9$Sdq+1))
pan.txf9$Sal=((pan.txf9$Sal)^2)
pan.txf9$mea=(1/sqrt(pan.txf9$mea))
pan.txf9$meh=(log(pan.txf9$meh))
pan.txf9$medf=((pan.txf9$medf)^8)
```

#Check (and remove NAs)

```
is.na(pan.txf9)
pan.txf9 <- na.omit(pan.txf9)
```

Inspect data before run further analyses

Kaiser-Meyer-Olkin measure of sampling adequacy

```
res=paf(object=as.matrix(pan.tx[, parameters]))
res$KMO                                           # KMO reveals 0.80654
```

according to Budaev [1] a FA is justified if KMO > 0.5, and if KMO < 0.7 the FA needs to be interpreted with caution

Prior the FA, run PCA and check the loadings

```
pca.pan.tx <- prcomp(pan.tx[, parameters], scale.=T, center=T)
pca.pan.tx.sum <- summary(pca.pan.tx)
pca.pan.tx.rot <- pca.pan.tx$rotation[,pca.pan.tx.sum$sdev>=1]          #show all PC's with Eigenvalues ≥ 1
```

2 PCs are justified (Eigenvalues ≥ 1), however loadings of the parameters on those PCs are not optimal. They should ideally be close to either -1 or 1, respectively.

Run a Factor Analysis with varimax rotation

```
fa.rot=factanal(x=pan.tx[, parameters], factors=2, rotation="varimax", scores="regression", lower=0.01)
fa.rot$loadings
as.table(fa.rot$loadings)
```

Extract factor scores

```
scores <- as.table(fa.rot$scores)
write.xlsx2(scores, file="Scores_season.xlsx", col.names=TRUE, row.names=FALSE)
```

add factor scores to data frame

```
scores.season <- c.bind(pan, scores)              # this step is needed because we need to align the scores
                                                  with the particular season
```

Run the post-hoc tests in order to explore significant variations between groups

```
disc <- aov(Factor1 + Factor2 ~ SEASON, data=scores.season)
TukeyHSD(disc)                                    # tests for significance of the different groups
```

References

1. Budaev S V. Using Principal Components and Factor Analysis in Animal Behaviour Research: Caveats and Guidelines. Ethology. 2010;116(5):472–80. 10.1111/j.1439-0310.2010.01758.x

2. Elle O. Einführung in die multivariate Statistik für Feldornithologen: Hauptkomponentenanalyse, Diskriminanzanalyse und Clusteranalyse. Vogelwarte. 2005;43:19–38.

3. Stuhlträger J, Schulz-Kornas E, Wittig RM, Kupczik K. Ontogenetic Dietary Shifts and Microscopic Tooth Wear in Western Chimpanzees. Front Ecol Evol. 2019;7:298. 10.3389/fevo.2019.00298
